# Prevention and management of critical care complications in cardiogenic shock: a narrative review

**DOI:** 10.1186/s40560-023-00675-2

**Published:** 2023-07-06

**Authors:** Jin Kirigaya, Noriaki Iwahashi, Kengo Terasaka, Ichiro Takeuchi

**Affiliations:** 1 Advanced Critical Care and Emergency Center, Yokohama City University Medical Center, 4-57 Urafune-Cho, Minami-Ku, Yokohama, 232-0024 Japan; 2 Division of Cardiology, Yokohama City University Medical Center, Yokohama, Japan

**Keywords:** Cardiogenic shock, Cardiac intensive care unit, Complication, Acute myocardial infarction

## Abstract

**Background:**

Cardiogenic shock (CS) is a common cause of morbidity and mortality in cardiac intensive care units (CICUs), even in the contemporary era.

**Main text:**

Although mechanical circulatory supports have recently become widely available and used in transforming the management of CS, their routine use to improve outcomes has not been established. Transportation to a high-volume center, early reperfusion, tailored mechanical circulatory supports, regionalized systems of care with multidisciplinary CS teams, a dedicated CICU, and a systemic approach, including preventing noncardiogenic complications, are the key components of CS treatment strategies.

**Conclusions:**

This narrative review aimed to discuss the challenges of preventing patients from developing CS-related complications and provide a comprehensive practical approach for its management.

## Background

In emergency cardiovascular care, the high mortality rate associated with cardiogenic shock (CS) remains a challenge [1–3]. Despite growing interest, mechanical circulatory support (MCS) has not been found to improve prognosis in randomized control trials (RCTs). Early reperfusion therapy is the only established method of improving prognosis in cases of CS associated with acute myocardial infarction.

Patients with CS are inherently susceptible to many complications related to the severity of the underlying cause of the CS and such complications increase mortality [1, 3, 4]. Therefore, a more comprehensive approach is needed to prevent CS-related complications and improve prognosis. However, more guidance is needed to support the development of the best practices specific to CS. Recent studies have suggested that treatment in high-volume centers, standardized treatment algorithms, dedicated care in cardiovascular intensive care units (CICUs), multidisciplinary therapy, and a systemic approach effectively prevent and treat CS-related complications and improve outcomes [2, 4–7].

Here, we aimed to discuss the challenges in preventing patients from developing CS-related complications and present a comprehensive practical approach for its management.

## Main text

### Systems of care in CS

Establishing an appropriate system of care can prevent and treat complications of CS. Representative elements of establishing a system of care are dedicated CICUs, the shock team approach, and treatment in a high-volume center.

#### Dedicated CICUs

CICUs with sophisticated and specialized equipment and sufficient medical staff to manage critical cardiovascular diseases are essential in managing CS diversity. The treatment at a dedicated CICU was associated with reduced mortality rates in patients with cardiovascular diseases requiring critical care [8–10]. Several reasons for better outcomes in dedicated CICUs have been reported. A retrospective observational study, in which patients admitted to the CICU were divided into either a low-intensity management group (*n* = 616) or a high-intensity management group (*n* = 1815), demonstrated that high-intensity staffing was associated with a lower CICU mortality rate in the matched cohort of patients (7.5% vs. 3.7%; odds ratio [OR], 0.53; 95% confidence interval [CI], 0.32–0.86) [8]. Another retrospective cohort study reported that transitioning from an open to a closed model was associated with a lower overall CICU mortality rate (OR, 0.63; 95% CI, 0.43–0.93) [10]. In that analysis, pre-specified interaction with an MCS device and unit model showed that treatment with such a device was associated with lower mortality rates in the closed model of a CICU (OR, 0.60; 95% CI 0.18–0.78). Thus, the beneficial effects of dedicated CICUs on the prognosis of CS have already been established, and future studies are needed to define the CICU settings appropriate for CS.

#### Multidisciplinary shock team approach

Given the time-sensitive nature of the complex medical, catheter-based, and surgical treatments used to care for patients with CS, some centers in Western countries have developed and implemented multidisciplinary “shock teams” comprising representatives from critical care cardiology, advanced heart failure, interventional cardiology, extracorporeal membrane oxygenation (ECMO), and cardiac surgery specialties [5, 11]. A previous study has reported that a “shock team” approach using a pre-established therapeutic protocol was significantly associated with reduced mortality. In one of the Inova Heart and Vascular Institute CS pathway [7] novel shock team approaches, physicians from various service lines were notified via a one-call “shock line” for consultation in cases of suspected CS. If CS was suspected, the interventional team would proceed with emergent coronary angiography, pulmonary artery catheter (PAC), and peripheral vascular evaluation for large-bore MCS access. Percutaneous MCS can then be considered if hemodynamic criteria are met. CS is evaluated daily by shock teams who perform PAC, echocardiography, neurovascular assessment, and end-organ perfusion studies. In a study of 204 consecutive patients with CS, a team-based approach reduced CS mortality from 76 to 47%. This finding was confirmed in a multicenter observational study. A report from the multicenter network of CICUs in North America compared shock management and CICU mortality among centers with and without shock teams [11]. Centers with shock teams used PAC more frequently (60% vs. 49%), MCS less frequently (35% vs. 43%), and more advanced types of MCS (53% vs. 43%) than those without shock teams. A shock team was independently associated with a lower CICU mortality rate (23% vs. 29%). The authors indicated that the involvement of a shock team may have allowed for the proper interpretation of PAC results and the appropriate selection of MCS.

Although it may not be possible to introduce foreign shock team protocols in Japan because of the difference in hospital sizes between Japan and Western countries, the benefits of the shock team approach have been established and should be implemented in our country.

#### Transportation to high-volume CS centers

Several studies have reported the clinical benefits of treatment in high-volume centers [1, 6, 12]. A shortened time to reperfusion is considered critical to the hospital volume–outcome relationship for acute myocardial infarction complicated by cardiogenic shock (AMICS) because early revascularization is the only established treatment for AMICS reducing mortality [13, 14]. The US Nationwide Inpatient Sample (NIS) administrative database study showed that based on a comparison of quartiles of annual hospital volume of CS treatment, patients in the highest volume quartiles (≥ 107 cases/year) were significantly more likely to receive early revascularization within 24 h of admission (34% vs. 17%) than patients in the lowest volume quartile (< 27 cases/year) [6]. However, early revascularization alone cannot explain the low mortality rates at high-volume centers. In an analysis of the outcomes of 15,259 patients with CS in the US, a steady increase in survival was noted with increased institutional experience using Impella devices and hemodynamic monitoring in patients with AMICS [15]. These studies indicate that the proper interpretation of PAC results and the appropriate selection and use of MCS in high-volume centers may contribute to improved prognosis. Future studies are expected to clarify the evaluation criteria for high-volume centers suitable for CS treatment, as each previous study used a different definition of a high-volume center.

In summary, the first step in preventing complications of CS and improving outcomes requires the establishment of a system of care, such as a dedicated closed CICU, a multidisciplinary approach, and treatment in a high-volume center.

## Acute phase in CICU

### Hemodynamic monitoring

Multiparametric hemodynamic evaluation with comprehensive invasive and non-invasive hemodynamic monitoring provide appropriate treatment and prevent critical care complications in CS. Here, we will discuss PAC, echocardiography, and lactate levels, which are the commonly used parameters for hemodynamic monitoring in the CICU.

#### PAC

Hemodynamic parameters obtained via PACs are essential for decision-making during the selection, initiation, titration, and weaning off vasoactive drugs and/or MCS support in patients with CS [16].After earlier RCTs failed to identify a significant improvement in clinical outcomes for patients with heart failure using a PAC [17], the utilization of PACs has notably declined in CICU patients [18, 19]. However, a recent meta-analysis of observational studies indicated that the use of PACs for CS may improve the outcomes [20]. This indicates the clinical utility of PAC in patients with CS in contemporary CICU settings.

The effectiveness of PACs can be maximized via several means. First, a complete assessment is required. The retrospective study involving over 1400 patients with CS from eight tertiary care centers evaluated three different approaches during the index hospitalization. These approaches included complete (42%) or partial (40%) PAC evaluation, or no invasive evaluation (18%) [20]. Complete hemodynamic profiling with PAC required the documentation of five parameters: right atrial pressure, pulmonary artery systolic pressure, pulmonary artery diastolic pressure, pulmonary capillary wedge pressure, and pulmonary artery oxygen saturation. The complete-PAC-evaluation group had the lowest in-hospital mortality compared with those in the other groups across patients with all Society for Cardiovascular Angiography and Interventions stages. Thus, PACs should be completely assessed when they are used as a hemodynamic monitor in CS treatment.

Second, staff in the CICU must possess proficiency in PAC monitoring, particularly during the initiation, titration, and weaning off MCS, with a specific emphasis on devices such as Impella, which play a critical role in the supportive care of patients with CS. Cardiac power output (CPO), calculated as the mean arterial pressure cardiac output/451, is an important index of hemodynamic recovery and a strong predictor of mortality in CS [21]. An increase in CPO can be helpful during the MCS weaning process. Moreover, PAC-derived indexes of right ventricular function, such as the pulmonary artery pulsatility index, can be useful in CS treatment with MCS [21]. Echocardiography is also a useful hemodynamic monitoring tool for CS during MCS treatment. However, it cannot replace PAC. In patients supported by MCS devices such as the Impella, the mechanical noise generated by the device and the continuous flow can potentially affect the accuracy of the most commonly used Doppler-based measurements. Furthermore, in patients with severe left ventricular dysfunction, the most common biplane left ventricular ejection fraction (LVEF) measurement is insufficient for detecting myocardial recovery or for guiding weaning strategies. Thus, hemodynamic monitoring using a PAC remains a valuable tool for patients with MCS, and it provides information that cannot be replaced by other monitoring methods. Healthcare professionals must develop proficiency in PAC monitoring during MCS utilization.

Third, the potential complications associated with PAC insertion must be considered to maximize the effectiveness and safety of PAC monitoring. The most frequent complications are reportedly related to the catheter insertion site (up to 3.6%) and strictly depend on the specific center’s experience [22]. Rarely, severe complications, such as heart block (0.3–3.8%) and pulmonary artery rupture (< 0.1%), may occur [22]. Additionally, a catheterization duration of longer than 4 days significantly increases the risk of PAC colonization (OR, 9.81; 95% CI, 1.24–77.5) [23]. The PAC should be removed immediately after the CS resolves to prevent these complications.

#### Echocardiography

Echocardiography allows the rapid evaluation of biventricular function and identification of severe valvular, pericardial, and large-vessel diseases or mechanical complications, aiding in adequate etiological treatments [16]. Echo-derived hemodynamic estimators have prognostic implications, even in critical care settings. A recent retrospective observational study has demonstrated that a low stroke volume index (< 35 mL/m^2^) and high E/e′ ratio (> 15) demonstrated the strongest association with hospital mortality [24]. Echocardiography is also useful in determining MCS withdrawal. Previous studies also proposed a lower LVEF (20–25%) and velocity time integral (10 cm/s) to ensure successful weaning from venoarterial ECMO (VA-ECMO) [25, 26].

However, ultrasound windows may not always be permissive in clinical practice, particularly among mechanically ventilated patients in the supine position [16]. In addition, image quality can be relatively poor in obese patients [27]. For instance, diameter of the inferior vena cava and its respiratory variations, generally used to determine right atrial pressure, may not be obtained in up to 22% of CS cases [27]. Furthermore, the echocardiographic estimation of pulmonary hypertension is less accurate than that by a PAC [28, 29].

#### Blood lactate levels

Blood lactate levels are also excellent markers of the hemodynamic status of CS. In a post hoc analysis of the Dobutamine Compared to Milrinone in the Treatment of Cardiogenic Shock randomized double-blind controlled trial, lactate clearance was a strong and independent predictor of in-hospital survival in patients with CS [30]. In a meta-analysis of studies comparing lactate clearance between survivors and non-survivors of CS, the median lactate clearance at 6–8 h was 21.9% (14.6–42.1%) in survivors and 0.6% (3.7–14.6%) in non-survivors. At 24 h, the median lactate clearance was 60.7% (58.1–76.3%) in survivors and 40.3% (30.2–55.8%) in non-survivors [31]. Furthermore, a recent multicenter RCT has demonstrated that the immediate implementation of the VA-ECMO strategy did not improve clinical outcomes compared with that by an early conservative strategy [32]. In this study, because of the worsening of the hemodynamic status, defined as a rise in serum lactate levels by 3 mmol/L above the lowest value during the past 24 h, ECMO bailout was used in 39% of the patients randomized to the conservative strategy group. The study’s primary endpoints—death from any cause, resuscitated circulatory arrest, and use of another mechanical circulatory support device at 30 days—did not differ between the immediate versus conservative groups (63.8% vs. 71.2%; *P* = 0.21). These results suggest that lactate level measurement is an established method of hemodynamic monitoring in patients with CS.

In summary, PAC, echocardiography, and blood lactate levels are useful parameters for CS treatment. However, each method has its own advantages and disadvantages, as described above. Whenever possible, multiple monitoring methods should be utilized to determine the degree of circulatory failure.

### Respiratory care

CS is often complicated by respiratory failure, and more than 50% of patients with CS require respiratory support [33, 34]. Therefore, appropriate management of respiratory failure in patients with CS is essential to prevent further complications.

#### Tracheal intubation for CS

The indication for tracheal intubation in patients with CS is often difficult to determine because clear criteria are lacking. However, intubation should be performed without hesitation if needed, because delays in mechanical ventilation initiation have been associated with mortality [35].

The complication rate during tracheal intubation is reportedly 22–54% in the general population [36, 37]. A drop in blood pressure is the most frequent complication [36], and shock is an independent risk factor of emergent endotracheal intubation-related severe hypotension and cardiac arrest [38]. Considering the high risk of hemodynamic collapse during intubation, agents with a reduced cardiovascular depressant action, such as benzodiazepine, should be considered in addition to reducing sedative dosage [39, 40]. In patients with tenuous hemodynamics, pursuing an awake intubation technique by an expert in airway management may prevent further decompensation; however, this approach has not been evaluated systematically.

Patients with airways that require more than two attempts or > 10 min to place a tracheal tube are conventionally defined as having difficult airways [41]. A difficult airway can lead to severe complications that can be fatal in patients with CS, including aspiration of gastric contents, hypoxemia, and cardiac arrest [39]. Thus, in patients with CS, the prediction of a difficult airway, successful single intubation by an experienced physician, and administration of low-dose sedatives are essential to prevent intubation-related complications.

#### Mechanical ventilation (MV) strategy

When positive pressure ventilation (PPV) is initiated, the gradient between the right atrium and abdominal compartment decreases, reducing venous return and right ventricular (RV) stroke volume [40]. PPV positively impacts non-shock congestive heart failure by reducing the preload. These variations must be considered in patients with RV dysfunction, where the RV is even more preload-dependent. PPV may lead to an abrupt reduction in venous return and worsen the hemodynamics in patients with CS and RV dysfunction [40]. The optimal positive end-expiratory pressure (PEEP) strategy has not yet been reported. However, in patients with RV dysfunction, the PEEP should be precisely adjusted to avoid hemodynamic compromise due to excessive reduction in venous return or increased RV afterload due to an increased PEEP [40].

The optimal tidal volume strategy has also not yet been reported. If invasive ventilation is required, lung-protective ventilation (6–8 mL/kg) should be performed to prevent pulmonary injuries [42]. Low tidal volumes optimize the blood flow between the pulmonary and parenchymal vasculatures. The decreased resistance in the pulmonary circuit lowers the stress on the RV versus higher tidal volumes. High tidal volume may induce ventilator-associated lung injuries characterized by inflammation, hyaline membrane formation, and increased vascular permeability [4]. Therefore, a low-tidal volume strategy is recommended for mechanical ventilation in patients with CS [43].

#### Extubation

The discontinuation of PPV rapidly increases venous return and sympathetic hyperactivity due to a catecholamine surge, increases heart rate and blood pressure, decreases oxygen supply, and increases the work of breathing. Therefore, pre- and post-loading adjustments and titration of the heart rate should be administered to patients with CS before extubation.

Although reports on CS are lacking, recent clinical guidelines for liberating patients at risk for extubation failure recommend using noninvasive preventive ventilation immediately after extubation [44, 45]. Noninvasive ventilation after planned extubation might be beneficial in CS cases in which the hemodynamic and respiratory status changes with extubation should be minimized.

In summary, respiratory management, including intubation, MV support, and extubation, may cause several complications in patients with CS. Because these complications can be fatal, prevention is essential. Evidence on respiratory management in patients with CS is lacking, and further comprehensive research is needed.

### Appropriate sedation

No specific drug is more efficacious in maintaining sedation in patients with CS requiring MV; therefore, sedative selection can be tailored to the patient’s clinical and hemodynamic characteristics.

#### Analgesia

Opiates have been recommended as the first-line analgesics and sedatives in critically ill cardiac patients, given their minimal effect on contractility and afterload and the potential to decrease myocardial oxygen demand [46]. No study has described the efficacy of one opioid over another. However, morphine should be avoided because it can induce venodilation and pharmacodynamic interactions with antiplatelet agents [47].

#### Sedation

Propofol can be considered an appropriate first-line sedative in patients with cardiovascular diseases requiring MV, considering its short half-life and the risk of delirium with benzodiazepines. However, propofol must be used with caution in patients with CS considering its negative effects on cardiac output via increased vasodilation, sympatholytic effects, and bradycardia necessitating higher doses of catecholamines [48, 49]. A small randomized trial including 59 patients treated with therapeutic hypothermia after cardiac arrest compared propofol/remifentanil to midazolam/fentanyl and reported that patients treated with propofol required more catecholamines [50]. In such cases, using alternative agents, such as benzodiazepines, may be appropriate.

Benzodiazepines may be considered an alternative in patients with CS and hemodynamic instability [34]. Due to the lack of evidence regarding the choice of a sedative agent for patients with CS, 2016 ESC guidelines of heart failure advise caution while using propofol due to its potential cardio-depressive side effects and recommend midazolam for those patients without citing any study as a reference [51]. However, given the association between benzodiazepines and delirium and their demonstrated association with increased mortality, they should be avoided if possible and considered second-line agents for maintaining sedation in patients at a lower risk of delirium [52].

Dexmedetomidine is an attractive option because of its ability to induce only light sedation without respiratory suppression. However, the potential side effects like hypotension and bradycardia are worrisome to patients with CS [53]. RCTs comparing dexmedetomidine and midazolam in the ICU showed that patients who were administered dexmedetomidine had more hypotension (20.6% vs. 11.6%) and bradycardia (14.2% vs. 5.2%) than those who were administered midazolam [53]. Bradycardia and hypotension may be potentially fatal complications of CS, and dexmedetomidine should not be used for first-line treatment.

In summary, propofol should be used as a first-line sedative drug for CS patients, and using alternative agents, such as benzodiazepine, may be appropriate in cases of hemodynamic instability.

### Bleeding complications

Preventing bleeding complications is important in managing CS to ensure optimal patient outcomes. The most common complications are gastrointestinal (31.5%) and vascular access site (23.8%) bleeding [54]. In a post hoc analysis of the CULPRIT–SHOCK trial, 21.5% patients with AMICS experienced at least one bleeding event up to 30 days, and bleeding increased mortality (hazard ratio [HR]: 2.11; 95% CI 1.63–2.75) [55]. The Japan Acute Myocardial Infarction Registry report demonstrated that major in-hospital bleeding was observed in 14.6% patients with AMICS, and in-hospital bleeding was independently associated with all-cause mortality (HR: 1.70; 95% CI 1.08–2.69) [56].

Patients with CS can easily bleed due to several reasons. First, treatment with MCS is major risk factor for bleeding [57]. In addition to the need for significantly larger access sheaths, the risk of bleeding may be increased by both consumptive coagulopathy and acquired platelet dysfunction in the setting of high shear stress in the case of MCS use [58]. Second, primary percutaneous coronary intervention (PCI), essential for improving AMICS prognosis, needs dual antiplatelet therapy, which induces bleeding complications. Third, critical end-organ hypoperfusion due to CS may cause disseminated intravascular coagulation and increase bleeding risks [43].

Bleeding complications can be prevented by taking appropriate preventive measures. First, femoral artery access should be avoided to prevent vascular access site bleeding complications. Some observational studies and a meta-analysis reported the advantage of radial access to reduce mortality and bleeding complications even in cases requiring an MCS [59, 60], although it may be challenging in hypotensive patients with CS. Second, monitoring of the use of anticoagulants is essential. In addition to continuous anticoagulant use with high activated clotting time (ACT) or activated partial thromboplastin clotting time goals (APTT), increased destruction of platelets can increase the risk of bleeding in patients with MCS [60]. Close monitoring and titration of ACT or APTT goals for anticoagulation are essential in patients at risk of bleeding complications with any MCS. Third, CICU staff should observe the vascular access site carefully. Most vascular site bleeding complications occur within 48 h of presentation [61]. Therefore, the puncture site needs to be frequently observed during this period.

Gastrointestinal bleeding is one of the most common bleeding complications [54]. Stress ulcers, defined as ulcers of the upper gastrointestinal tract that occur due to illness during hospitalization, are common in the CICU setting [2]. The report from the NIS database revealed that CS was significantly associated with gastrointestinal bleeding (OR: 8.34; 95% CI: 8.19–8.49, *P* < 0.001) [62]. Therefore, stress ulcer prophylaxis with a proton pump inhibitor is reasonable for patients with CS, although the data supporting this approach are lacking [63].

## Recovery phase in CICU

### Prevention of altered mental status

Although there are few studies on altered consciousness in patients with CS, it has been reported as a poor prognostic factor in critically ill patients [64, 65]. A sub-study of the CardShock Study, a multicenter prospective observational study that investigated the clinical perspective and outcome of CS and developed a risk prediction score for short-term mortality, reported that altered mental status was detected in 68% of patients with CS, and 90-day mortality was significantly higher among patients with altered mental status (51% vs. 22%, *P* < 0.001) [65]. The following characteristics are associated with an increased risk of delirium and should be screened in all patients admitted to the CICU [66]: old age, history of cognitive impairment/delirium in previous hospitalizations, history of heart failure, polypharmacy, history of drug and alcohol abuse, cardiac arrest, MCS use, and invasive mechanical ventilation.

Although CS-specific methods have not been reported for preventing altered consciousness or delirium, a systematic approach to delirium prevention is required. Pharmacological therapies have demonstrated mixed results with questionable efficacy, and the routine administration of antipsychotic drugs should be avoided [67]. The evidence-based strategy to prevent delirium is referred to as the ABCDE bundle: awakening and breathing coordination, delirium monitoring/management, and early exercise/mobility [68]. The pathophysiology of CS is complex, and it is difficult to implement an ABCDE bundle uniformly. However, previous studies demonstrated the benefits of this bundle for general diseases, and it should be offered as a delirium prevention strategy for CS.

### Early enteral nutrition (EN)

The nutritional strategies for CS remain controversial. Early EN within 48 h of admission is recommended for most critically ill patients [69, 70]. However, these guidelines advise that delayed EN should be considered in patients with uncontrolled shock and failure to achieve the hemodynamic and tissue perfusion targets.

When initiating EN for patients with CS, the effects of low output on organ perfusion and catecholamine levels should be considered. The compensatory function of circulatory failure causes hypoperfusion of the intestinal tract and several complications, including decreased intestinal peristalsis and absorption, nonocclusive mesenteric ischemia, and ischemic enterocolitis [71]. Furthermore, prolonged fasting due to CS can cause bacterial translocation via atrophy of the epithelial mucosa of the small intestine, resulting in gastrointestinal problems [72]. Additionally, catecholamine use should be considered when initiating EN in patients with CS. Norepinephrine and epinephrine can effectively improve systemic vascular resistance, thus maintaining perfusion pressure in the brain and heart. However, they can exacerbate bowel ischemia. A previous study reported that high-dose noradrenaline (≥ 0.5 μg/kg/min) was associated with digestive complications [73]. Dobutamine use needs consideration when initiating EN in patients with CS. A post hoc analysis of the NUTRIREA-2 trial, in which ventilated adults with shock were randomly assigned to receive EN or parental nutrition, showed that dobutamine use was significantly associated with acute mesenteric ischemia [74].

Therefore, the decision to initiate EN in patients with CS is often difficult. However, a recent study using a national inpatient database in Japan reported that early EN was associated with reduction in mortality in patients with CS who underwent ventilation and were administered low- or medium-dose noradrenaline (< 0.3 μg/kg/min) [75]. Another study reported no significant correlations between EN tolerance, vasoactive drug doses, or blood lactate levels in patients with CS [76]. These studies suggested the benefit of introducing EN in patients with CS treated with catecholamine.

The appropriate EN for patients with CS undergoing VA-ECMO remains unknown. Previous studies reported feeding intolerance in 20–45% of patients receiving EN during VA-ECMO [77, 78]. Uncertainty about nutrition in ECMO may occur, because using VA-ECMO traditionally involves paralysis and/or heavy sedation, which may affect gut function. In addition, the effect of VA-ECMO itself has been reported to reduce gut perfusion [77]. Furthermore, using huge volumes of intravenous fluid to maintain high flow rates can cause edema, reducing gut absorption and motility [77]. However, early EN may improve prognosis, even in patients with CS undergoing VA-ECMO treatment. A retrospective study based on the Japanese Registry of All Cardiac and Vascular Diseases Diagnosis Procedure Combination (JROAD–DPC) database reported that early EN is associated with lower mortality in patients with CS or obstructive shock requiring at least 2 days of VA-ECMO treatment [79]. However, in this study, only 12% of patients undergoing VA-ECMO received early EN; the results of this observational study require further validation.

In summary, it is difficult for patients with CS to determine when and how to start EN due to the lack of evidence. Recently, it has been reported that a multidisciplinary approach, including nutritionists and developing protocols for nutritional administration, contributes to improved prognosis in critically ill patients [80]. Therefore, a multidisciplinary team, including a nutritionist, may be better to discuss the appropriate time to initiate EN in CS patients, considering individual patient conditions.

### Early mobilization

Early mobilization for patients with CS can be challenging, and evidence of its application is scarce. Prolonged bed rest in critical-care patients contributes to several short- and long-term complications [81, 82], including ICU-acquired weakness, neuromuscular weakness, reduced quality of life, hospital re-admission, and death. Although the evidence regarding the benefit of systematic early mobilization remained inconclusive, some data suggest that early mobilization of patients in the ICU may reduce the length of hospital stay and improve physical function after hospital discharge [83, 84].

However, several CS-specific issues make it challenging to decide when and how to initiate mobilization in patients with CS. First, the contraindications of early mobilization in patients with CS exist. Absolute contraindications to mobilization typically include active myocardial ischemia, hemodynamic instability, deterioration of pulmonary congestion, or uncontrolled bleeding [85]. It is completely unsafe to initiate mobilization in such cases. Second, vasoactive drugs are one of the most common patient-related barriers to early mobilization in the ICU [86, 87]. High doses of vasoactive agents can induce sudden changes in blood pressure and heart rate during movement. Third, using MCS may present a barrier to early mobilization in patients with CS [21]. Mobilization in patients on percutaneous MCS is challenging for clinicians who must manage the risk of pump dislodgement or malfunction in patients dependent on extracorporeal flow. These CS-specific issues make it challenging to implement early mobilization in patients with CS.

Thus, deciding the optimal timing to initiate mobilization in patients with CS is challenging. However, several studies suggest that early mobilization may improve outcomes and reduce hospital stays for patients with CS. Patients with MCS, especially with an axillary Impella, have been reported to initiate early mobilization safely. Another study demonstrated that even in patients undergoing ECMO with femoral cannulation, mobilization was safe and feasible in centers with specialists having sufficient expertise and training [88].

Ultimately, the interprofessional team should decide whether mobilization can be initiated in a patient with a CS based on a careful assessment of the patient’s condition and needs. Monitoring the patient's vital signs and MCS parameters during the mobilization process is essential [89]. Discontinuing the therapy session is reasonable if significant neurological, cardiovascular, or respiratory derangements occur.

### Preventing infections

Nosocomial infections have been observed in 20–30% patients with CS [3, 90, 91]. The strongest association of developing a nosocomial infection was observed with increasing low output syndrome [91]. A recent study using the NIS database showed that the most common nosocomial infection was urinary tract infection (9.2%), followed by hospital-acquired pneumonia (HAP) (6.8%), central line-associated bloodstream infections (CLABSI) (1.5%), bacteremia (1.5%), skin-related infections (1.5%), and *Clostridium difficile* infection (1.3%) [91]. Increased mortality risk was observed among patients with nosocomial infections (adjusted OR: 1.11, 95% CI: 1.07–1.16), especially among those with sepsis-associated nosocomial infections compared with those without sepsis (adjusted OR: 2.95; 95% CI 2.72–3.20). Therefore, physicians treating patients with CS should be aware of infections as a complication of CS. Here we describe three infections that are common in patients with CS.

#### Respiratory tract infections

Respiratory tract infections are one of the most common infection types in patients with CS, because more than half of all patients with CS develop progressive respiratory failure requiring invasive mechanical ventilatory support, the leading cause of ventilator associated pneumonia (VAP) [91], a subtype of HAP that is defined as pneumonia that develops more than 48–72 h after endotracheal intubation [92]. A study using the NIS database reported that HAP occurred in 6.8% of patients with CS [93]. The risk factors for HAP are generally patient-related (male sex, pre-existing pulmonary diseases, or multiorgan system failure) or treatment-related (intubation or enteral feeding) [94].

The ventilator bundle is an effective method for preventing VAP in CICUs [94]. The ventilator bundle comprises head of bed elevation, sedation protocols targeting light sedation, daily sedation vacation, oral chlorhexidine rinse, and endotracheal tube with subglottic secretion drainage [93, 94].

Once HAP is confirmed, recent guidelines recommend considering local antibiotic resistance patterns and patient risks for resistant pathogenic infections when formulating an initial empiric antibiotic regimen [94, 95]. Consultation with an infectious disease specialist should be considered. This strategy is expected to manage all CS-associated infections, including respiratory infections. A systematic infection disease specialist consultation program may improve the appropriateness of antimicrobial therapy prescribed in the CICU and adherence to local antibiotic therapy guidelines [96].

#### Catheter-associated urinary tract infection (CAUTI)

CAUTI was reported to be the most common nosocomial infection in patients with CS (9.2%) in a study using data from the NIS database [91]. The risk factors for developing CAUTI are the duration of catheterization, female sex, older age, diabetes mellitus, bacterial colonization of the drainage bag, and errors in catheter care [97].

The CAUTI bundle is an effective method for CAUTI prevention. The CAUTI bundles comprise processes for insertion and maintenance of Foley catheters, indications for indwelling Foley catheters, appropriate testing for CAUTIs, alternatives to indwelling devices, and sterilization techniques. Implementation of the CAUTI bundle reduces CAUTI incidence in critically ill patients [97, 98].

#### Bloodstream infection (CLABSI and non-CLABSI)

In patients with CS, bloodstream infections (CLABSIs and non-CLABSIs) can be caused by monitoring or therapeutic devices such as PAC, central venous lines, arterial lines, ECMO, Intra-aortic balloon pump (IABP), and Impella. Bloodstream infection is associated with higher hospital costs, longer stay, and potentially higher mortality [99]. Risk factors for bloodstream infections include host factors (e.g., chronic illness, immunodeficiency, malnutrition, and age) and catheter-related factors (e.g., duration of catheterization, type of catheter, conditions of insertion, access site care, and skill of catheter inserter) [99]. The incidences per 1000 catheter days for each catheter type were as follows: peripheral venous catheters, 0.5; peripherally inserted central venous catheter, 1.1; central venous catheter (CVC), 1.6; arterial catheters, 1.7; and PAC, 3.7 [100]. In therapeutic temporary MCS, the reported infection rates for IABP, ECMO, and Impella were 0.5–35%, 3.5–17.7%, and 1.1%, respectively [101].

When using intravascular catheters, including CVCs, arterial lines, or percutaneous MCS devices, a multicomponent central-line bundle should be implemented to reduce the risk of CLABSIs [102]. Bundles of prevention strategies for central venous bloodstream infections have been implemented frequently to reduce complications and improve the outcomes of critically ill patients [103]. The central-line bundles comprise maximum barrier precautions, chlorhexidine skin disinfection, optimal catheter site selection (avoiding the femoral approach), ultrasound-guided prominent line placement, and daily confirmation of line requirements. Although this bundle has not been specifically validated among CS cohorts, the American Heart Association scientific statement in 2020 suggested using this bundle for CS management to prevent bloodstream infections [2]. Early adequate antimicrobial therapy is essential in improving patient outcomes and should be based on guidelines and direct examination of available samples [57].

In summary, although data among patients with CS are scarce, implementing care bundle strategies to minimize infections has shown positive results in critically ill patients. Upon infection confirmation, local antibiotic resistance patterns, individual patient risks for resistant pathogenic infections, and consultation with an infectious disease specialist should be considered.

### Palliative care (PC)

Despite advances, CS continues to have a high burden of morbidity and mortality. Therefore, PC services play an important role in the management of selected patients with CS. However, PC for patients with CS poses several challenges that must be addressed.

Despite the integration of PC to cardiovascular care supported by major cardiology societies’ guidelines [104], PC is not implemented frequently in CICUs in the contemporary era. A recent retrospective study of the NIS found that only 4.5% of hospitalizations for AMICS used PC [105]. This suggests that barriers to implement PC in patients with acute cardiovascular disease exist. Previous studies have shown that integrating PC into CICUs is challenging, including physicians’ lack of knowledge and skills in PC, the perception that care in the ICU and PC are sequential and mutually exclusive, and unrealistic expectations of patients, families, and healthcare providers [105].

PC improves the quality of life and symptom burden in patients at various stages of cardiovascular diseases and should be considered along with curative treatments [106]. The 2017 Nationwide Readmissions Database demonstrated that PC services were associated with lower 30-day readmission rates and hospitalization costs. This may be because detailed discussions about prognosis and advanced planning with the PC team may have resulted in changes to patient care goals, leading them to consider less aggressive measures or hospice care after discharge.

To ensure PC access for all patients with CS, it is necessary to standardize primary PC competencies for CS and provide appropriate educational opportunities for all physicians involved in managing CS cases. A primary PC educational program for all physicians engaged in HF care was developed in 2017 with the objective to provide knowledge on PC for cardiovascular diseases. This educational program is called the HEart failure Palliative care Training (HEPT) program for comprehensive care providers [54]. Shibata et al. reported that physicians who completed the HEPT program showed significant improvements in practice, difficulty, and knowledge regarding in heart failure PC. Another effort to introduce PC services into the CICU is the PC bundle. Similar to previously mentioned bundles used in the ICU, such as the “ventilator bundle,” Nelson et al. developed and reported on the utility of a practical set of measures collectively termed as the “PC bundle.” [55]

In summary, although various efforts have been made, such as implementing educational systems and creating bundles, the implementation of PC for CS in the CICU is still insufficient due to its unique nature as mentioned above. Further research is needed to identify the right time and indication for PC referral for patients with CS.

Despite advances, CS continues to have a high burden of morbidity and mortality. Therefore, PC services play an important role in the management of selected patients with CS. However, PC for patients with CS poses several challenges that must be addressed.

## Conclusion

**Fig. 1:**
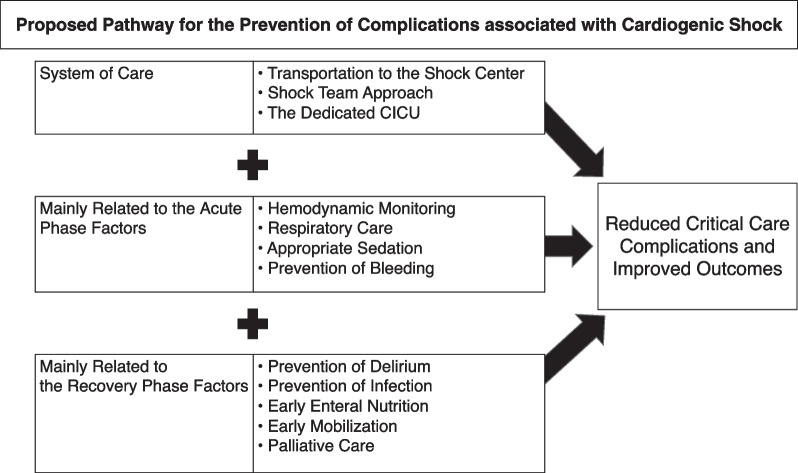
Proposed pathway for the prevention of complications associated with cardiogenic shock. *CICU* cardiac intensive care unit

Despite improvements in CS survival in recent years, morbidity and mortality rates remain high, and few evidence-based therapeutic interventions are known to improve patient outcomes. As discussed in this review, a comprehensive approach tailored to the CS phase (Fig. 1), may be key in preventing CS complications and improving prognosis. Future research through rigorous multicenter clinical trials and large prospective registries is required to better identify the epidemiology of CS-related complications and evaluate existing and new therapies.

## Data Availability

Not applicable.

## Abbreviations

ACT: Activated clotting time
CAUTI: Catheter-associated urinary tract infection
CI: Confidence interval
CICU: Cardiovascular intensive care unit
CLABSI: Central line-associated bloodstream infection
CPO: Cardiac power output
CS: Cardiogenic shock
EN: Enteral nutrition
ECMO: Extracorporeal membrane oxygenation
HR: Hazard ratio
HEPT: HEart failure Palliative care Training
IABP: Intra-aortic balloon pump
JROAD-DPC: Japanese Registry of All Cardiac and Vascular Diseases Diagnosis Procedure Combination
LV: Left ventricular
LVEF: Left ventricular ejection fraction
MSC: Mechanical circulatory support
MV: Mechanical ventilation
NIS: Nationwide inpatient sample
OR: Odds ratio
PAC: Pulmonary artery catheter
PC: Palliative care
PEEP: Positive end-expiratory pressure
PCI: Percutaneous coronary intervention
PPV: Positive pressure ventilation
RCTs: Randomized control trials
RV: Right ventricular
VA-ECMO: Venoarterial ECMO
VAP: Ventilator associated pneumonia
